# Biological Properties of Low-Toxic PLGA and PLGA/PHB Fibrous Nanocomposite Scaffolds for Osseous Tissue Regeneration. Evaluation of Potential Bioactivity

**DOI:** 10.3390/molecules22111852

**Published:** 2017-10-28

**Authors:** Boguslawa Żywicka, Izabella Krucińska, Jerzy Garcarek, Maria Szymonowicz, Agnieszka Komisarczyk, Zbigniew Rybak

**Affiliations:** 1 Department of Experimental Surgery and Biomaterials Research, Wrocław Medical University, Pasteura 1, 50-367 Wrocław, Poland; boguslawa.zywicka@umed.wroc.pl (B.Ż.); maria.szymonowicz@umed.wroc.pl (M.S.); zbigniew.rybak@umed.wroc.pl (Z.R.); 2 Department of Material and Commodity Sciences and Textile Metrology, Technical University of Lodz, Zeromskiego 116, 90-924 Lodz, Poland; izabella.krucinska@p.lodz.pl; 3 Department of General Radiology, Interventional Radiology and Neuroradiology, Wrocław Medical University, Pasteura 1, 50-367 Wrocław, Poland; jgarcarek@gmail.com

**Keywords:** biocompatibility, bioresorption, nonwoven fabrics, bone implant, poly(l-lactide-*co*-glycolide), synthetic poly([R,S]-3-hydroxybutyrate), encapsulated growth factor

## Abstract

The aim of the study was to evaluate the biocompatibility and bioactivity of two new prototype implants for bone tissue regeneration made from biodegradable fibrous materials. The first is a newly developed poly(l-lactide-*co*-glycolide), (PLGA), and the second is a blend of PLGA with synthetic poly([R,S]-3-hydroxybutyrate) (PLGA/PHB). The implant prototypes comprise PLGA or PLGA/PHB nonwoven fabrics with designed pore structures to create the best conditions for cell proliferation. The bioactivity of the proposed implants was enhanced by introducing a hydroxyapatite material and a biologically active agent, namely, growth factor IGF1, encapsulated in calcium alginate microspheres. To assess the biocompatibility and bioactivity, allergenic tests and an assessment of the local reaction of bone tissue after implantation were performed. Comparative studies of local tissue response after implantation into trochanters for a period of 12 months were performed on New Zealand rabbits. Based on the results of the in vivo evaluation of the allergenic effects and the local tissue reaction 12 months after implantation, it was concluded that the two implant prototypes, PLGA + IGF1 and PLGA/PHB + IGF1, were characterized by high biocompatibility with the soft and bone tissues of the tested animals.

## 1. Introduction

The purpose of an implant is to not only replace the damaged tissue but also to support the body in the process of regenerating the damaged area. In the case of implantable bone devices, there are two groups: active and inert products. Inert materials are those that, after implantation, do not integrate into the adjacent tissue. Consequently, over time, fibrosis of surrounding tissues occurs, as does degeneration and a loosening of the prosthesis. The term ‘active bone implant’ refers to products that fully integrate with the surrounding tissue. One method of activation of the implanted devices is to develop a porous structure, which allows the penetration of adjacent tissue cells into the implant and enables its osteointegration. There are a number of published studies indicating that a porous structure supports the processes of both the regeneration of the damaged area and the integration of the implant [1,2,3,4]. In particular, a porous structure allows osteoconduction and vascularisation, which is always important in cases of implants. Research carried out mainly on soft tissues indicates that, if the implanted material has a structure that reproduces the natural tissue, the process of healing and integration takes place more quickly [5,6,7]. Recently, a number of studies have been conducted on polymer-biological composite bone implant materials with engineered structures [8,9,10], which may be an alternative to xenogenic implants. In the manufacture of such implants, a number of textile techniques are used, including electrospinning and rapid prototyping and also techniques intended to produce porous materials with nonfibrous structures such as phase separation, the formation of foams, and other techniques [11,12,13,14,15]. In the majority of publications, emphasis is placed on the significance of the influence of the porous structure of materials for bone implantation and scaffolds in the process of tissue regeneration [16,17]. The materials for bone implantation, depending upon the manufacturing technique, are characterized by a porosity of up to 90%, with an average pore diameter of 1 μm to 800 μm [1]. At the same time, a porous structure with a pore size preferred for the proliferation of cells often results in the ‘fall out’ of cells from the scaffold. For this reason, a number of multilayer structures and composite models have been developed and are characterized by a different pore structure in each layer [3]. Another issue is the immobilization of bioactive substances in the structure of the material [12]. There is still a great need for drug delivery systems that allow for improved release kinetics of multiple growth factors or other compounds in order to enhance their therapeutic efficacy [18]. One of these is the local microsphere growth factor release system. The research has shown that a number of growth factors, promote tissue repair and regeneration, including bone formation; moreover, the efficacy of growth factor therapies in periodontology and implantology has been well characterized in a variety of in vitro and in vivo studies [19]. One of the recommended growth factors is bone morphogenetic protein (BMP), with known and accepted efficacy, but recent studies have shown that the interaction of BMP and insulin-like growth factor- 1 (IGF-1) was more beneficial in bone regeneration than the use of a single BMP [18]. The mechanisms of interaction and the role of IGF1 in cell and tissue metabolism are still being explored. Numerous IGF1 functions have demonstrated their effects on cell proliferation and differentiation, and on angiogenesis, which facilitates the overgrowth of the implant [20]. The clinical observations and animal data suggest that the essential pool of IGF1 in the bone matrix may not be sufficiently available for new bone formation during the aging process and in disease states. Therefore, the modulation of IGF1 deposition in the bone matrix could potentially be a therapeutic approach to delay or prevent bone loss [21]. Similarly, hydroxyapatite supplementation may be used to promote local regeneration.

Another important factor influencing the quality of the implant material for tissue regeneration is its resorbability. Among resorbable polyesters, polylactide (PLA) and its copolymers with glycolide (PLGA), as well as polyhydroxybutyrates (PHB), play a leading role in biomedical applications. An accelerated process of degradation occurs when the PLA used is atactic, built from heterochiral chains l,d-PLA. Additionally, another possibility of modification is copolymerisation with a glycolide repeat unit, which allows the production of the poly(lactide-*co*-glycolide) (PLGA) copolymer. All the mentioned polymers undergo degradation by hydrolysis over time, and the extent of degradation depends on the chemical composition, the presence of a biocatalyst, the pH level, and the temperature. Bioresorbability is a very desirable property of implanted materials destined for temporal contact with tissues because it can help prevent subsequent revisions, potential pain, and infections.

Reports on the biofunctionality of implantable devices based on PLA, PLGA, and PHB indicate that the impact is not always beneficial. After the clinical use of surgical nails using PLA in the knee, swelling was observed, and the patient required a surgical revision [22]. The same authors showed a severe reaction of the giant cells around the PLA materials. Adverse reactions were associated with changes in the crystallinity of the polymers in the biodegradation process. The positive impact and high biocompatibility of PLGA microparticles as carriers were also demonstrated [23]. Research is being conducted on new biodegradable polyesters that are synthesized using less toxic compounds. As an example, Dobrzynski et al. synthesised poly(lactide-*co*-glycolide) PLGA using Zr(AcAc)_4_ as an initiator [24,25,26,27]. 

Implantable medical devices can cause an immunologically mediated cutaneous reaction to a substance. The programmed bioactivity of PLGA and PLGA + PHB implants requires efficacy and safety testing, not only in vitro but also in vivo.

The aim of the present study is to assess the allergenicity and the local response of bone tissue after the implantation of two newly developed prototypes of fibrous implants for osseous tissue regeneration, which are characterized by high biocompatibility in vitro and by resorbability, based on PLGA polymers manufactured with a zirconium initiator. Alloplastic PLGA and PLGA/PHB implants with potential bioactivity by IGF1 and HAp supplementation were prepared.

## 2. Materials

**Figure 1:**
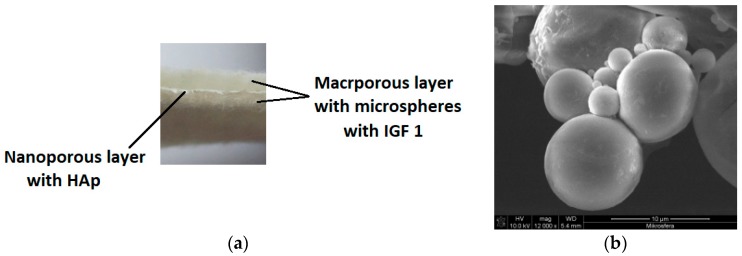
Construction of a multilayer prototype of the bone implants. (**a**) Structure of a multilayer nonwoven prototype; (**b**) Structure of microspheres with insulin-like growth factor-1 (IGF-1).

Two prototypes of bone implants were produced. The raw material that was used to produce the first test material was a copolymer of lactide and glycolide, designated as PLGA [26]. The second prototype was produced from a mixture of PLGA and poly(hydroxybutyrate), designated as PLGA/PHB. The amount of PHB in the mixture was 10% wt. Both designed implants had a multilayer structure with a dominant role for a nonwoven material with large pores, which had a mean diameter of 150 μm, forming a space for the the cell culture (Figure 1a).

Macroporous layers were produced by a card system, with the average surface mass at the level of 325 g/m^2^ and average thickness equal to 2.4 mm. Two layers with a pore size of several hundred micrometres were separated by another layer with nanometer pores of an average pore diameter of 10 to 17 nm; the role of those layers was to overgrow and maintain the cells in the material structure. The nanoporous layer was produced from the same polymer as the layers with large pores, with the addition of a 1% wt. solution of hydroxyapatite. The hydroxylapatite was in the form of nanoparticles delivered by Sigma Aldrich (St. Louis, MO, USA), with an average grain size of 200 nm. Moreover, the bioactivity of the proposed product was induced by introducing a biologically active agent: insulin-like growth factor IGF1 (Sigma Aldrich, USA). This active agent was encapsulated in calcium alginate microspheres (Figure 1b). The microspheres were formed from a 5% wt. solution of sodium alginate in biologically pure water, to which was added 25 μg IGF1 per 100 mL polymer solution. Microspheres were formed on a laboratory stand for that purpose and solidified in a 10 wt. % solution of calcium carbonate Ca_2_Cl·6H_2_O in biologically pure water. During the solidification, the replacement of the Na^+^ by Ca^2+^ ions in the alginate salt was observed. The decanted microspheres were filtered and then suspended in the biologically pure water. The suspension of the microspheres was filtered through the three-layer material. The activity of the IGF1 was confirmed during biological tests. Final products in the form of prototypes of the PLGA + IGF1 and PLGA/PHB + IGF1 implantable products were assessed from the point of view of their structure. Implants made from PLGA were characterised by a surface mass of 665.95 g/m^2^ and a thickness of 2.8 mm. This multilayer structure was characterised by an average pore diameter of 172,877.1 nm and a total pore area of 0.139 m^2^/g. The implantable material produced from PLGA/PHB + IGF1 had a surface mass equal to 692.34 g/m^2^ and a thickness of 2.7 mm. The porous structure of this material was characterised by an average pore diameter of 181,042.3 nm and a total pore area of 0.133 m^2^/g. Prototypes of the implantable products containing active insulin-like growth factor (IGF1) were tested on animals. As the control material, the same product was applied without IGF1. The Local Ethics Committee for Animal Experimentation in Wroclaw accepted the proposed and currently shown animal studies as admissible (consent No. 11/2008 and 88/2012, 89/2012, and 90/2012). For the implantation research, samples of the layered implant materials with dimensions of 3 × 3 × 6 mm were used. All materials were sterilized using a radiation dose of 28 kGy (before the IGF1 introduction) and a second dose of 15 kGy (after the IGF1 introduction into the fibrous implant). In the preliminary biological studies that were conducted to assess the potential risk of toxicity of the test materials, no cytotoxic or mutagenic activity was found in vitro. In animal model studies using both genders, there was no evidence of systemic toxicity after chronic implantation, the blood parameters were within the reference values, and the internal organs showed normal structures and functions. The IGF1 levels in the rabbits’ serum following the implantation of samples of PHB/PLGA + IGF1 and PLGA + IGF1 were normal.

## 3. Methodology

### 3.1. Sensitisation Test

A maximisation test using extracts (Guinea-Pig Maximisation Test, GPMT) was performed to assess the potential causes of delayed-type hypersensitivity for PLGA + IGF1 and PLGA/PHB + IGF1 implants [28,29,30,31]. 

The study was carried out on albino guinea pigs of both sexes with an average initial body weight of 355.68 g (300 to 430 g). The animals for testing came from a certified supplier of laboratory animals. During the experiment, the guinea pigs were kept in cages under controlled humidity (55% to 65%) and temperature (20 °C to 25 °C) and were given wholesome granulated food and water. with the addition of ascorbic acid in the amount of 200 mg/kg of their body mass, ad libitum.

For the preparation of the extracts, a saline solution was used. The extracts for testing were prepared with a proportion of a 1 g sample of PLGA + IGF1/10 mL + 10.1 mL of 0.9% NaCl and a 1 g sample of PLGA/PHB + IGF1/10 mL + 10.89 mL of 0.9% NaCl, taking into account their absorptivity. The extracts were incubated at 37 °C for 72 h.

After the acclimatisation period, the animals were randomly divided into three groups:

- The experimental group with PLGA + IGF1 has 10 animals,
- The experimental group with PLGA/PHB + IGF1 has 10 animals,
- The control group with 0.9% NaCl had five animals.

The day before the examination, during the intradermal induction phase, the guinea pigs’ fur was cut on the back in an area of 5 × 10 cm. Within each group of animals, each animal received three pairs of injections of 0.1 mL of the following solutions: the first pair of injections was comprised of a stable emulsion of complete Freund’s adjuvant, mixed 50:50 with physiological saline; the second pair of injections was comprised of an extract from the samples to be tested; and the third pair of injections was comprised of the extract of the tested samples emulsified in a volume ratio of 50:50 stable emulsion of complete Freund’s adjuvant and physiological saline.

The animals classified as the control group were also given three pairs of injections: the first pair was comprised of a stable emulsion of complete Freund’s adjuvant, mixed 50:50 with saline; the second pair was comprised of physiological saline; and the third pair was comprised of saline emulsified in a volume ratio of 50:50 by a stable emulsion of complete Freund’s adjuvant and solvent.

In the local induction phase, six days after the end of the intradermal induction phase, the fur was cut on the back of the animal again, and the injection site was checked. In the injection sites of extracts from the samples of PLGA + IGF1 and PLGA/PHB + IGF1, there was no skin irritation. In the nonirritated areas of skin, 10% sodium dodecyl sulphate was massaged.

After 24 h, the injection site was covered with filter paper and soaked with the tested extracts (experimental groups) or physiological saline (control group). Flakes of paper were secured with occlusive dressings (Blenderm 3M, 3M Poland Sp. z o.o., Kajetany, Poland). The dressings and paper flakes were removed after 48 h.

In the challenge phase, 13 days after the end of the local induction phase, all of the animals’ fur was cut on the right side. On day 14, in the cut-off space, the filter paper was applied and soaked with the tested extracts for the experimental group and with physiological saline solution for the control group. The tested areas were secured with Blenderm 3M dressings. The dressings and flakes were removed after 24 h.

### 3.2. Evaluation of Local Reactions after Implantation

The implantation research was done in four groups. These were two experimental groups of PLGA + IGF1 and PLGA/PHB + IGF1, control PLGA, and PLGA/PHB without microspheres and with IGF1.

### 3.3. Surgical Procedures

The tests were carried out on 24 New Zealand white rabbits of both sexes that had an average weight of 2.7 kg (± 200 g). The rabbits were kept singly in cages under controlled humidity (28% to 37%) and temperature (16°C to 20 °C). The animals had free access to water and were fed with a standard pelleted feed for rabbits (LSK), with an average daily consumption of between 50 to 70 g per rabbit. For each planned date of autopsy and all types of material tested, a minimum of three were rabbits were sacrificed, (five rabbits at later time points).

Beginning 24 h before the scheduled surgical procedures, the rabbits were subjected to fasting with access to water. An area of approximately 5 cm × 5 cm of the fur around the hip was removed mechanically. The rabbits were anesthetized with an intramuscular injection of anaesthetic mixture: Xylazine at a dose of 5 mg/kg and Ketamine at a dose of 35 mg/kg. Full analgesia was obtained 10 to 15 min after the injection and lasted for 60 to 80 min. The full effect lasted for 120 to 140 min. After complete analgesia, at the height of the hips, the skin was disinfected with SkinSept Color (Ecolab, St. Paul, MN, USA) and incisions of 4 to 5 cm long were made, running along the base of the proximal femur. Then, the lesser and the greater trochanter were exposed, in which two holes having a diameter of 3 mm and a length of 6 mm were drilled. In these cavities, the two test samples were placed on the right, and the control samples were placed on the opposite side. The muscles and soft tissues were closed with a single surgeon’s knot of MonoPlus 3-0 absorbable sutures (B Braun Medical Coe., Rubi, Spain). The skin was closed with a single stitch of Novosyn 2/0 (B Braun Medical Coe., Rubi, Spain).

In the postoperative period, the animals were housed in cages with free access to water and food under constant medical-veterinary care. The overall health of the rabbit was evaluated, with special emphasis on the healing of the surgical wounds, the active and passive mobility of the hip, and food intake [28,29,32]. 

### 3.4. Post Mortem Examinations

At the planned study time points of one, two, three, six, nine, and 12 months after implantation, euthanasia was performed on the rabbits by an intravenous injection of pentobarbital (trade name: Morbital, producer: Biowet, Pulawy, Poland) at doses of up to 80 mg/kg, administered in fractionated doses to achieve respiratory arrest and the cessation of heart function. Prior to pentobarbital administration, the general health status of the animals was assessed. During the autopsies, first, the postoperative wound was evaluated macroscopically, along with the appearance of the tissue at the implantation site. Afterward, the appearance of the selected internal organs was assessed. A macroscopic evaluation of tissue after the implantation of the PLGA + IGF1 and PLGA/PHB + IGF1 materials was carried out in relation to the tissues of the animals in the control group, which were implanted with PLGA and PLGA/PHB. For further radiological and histological studies, the femur bones were collected along with the implants.

### 3.5. X-ray Examinations

The X-ray examinations were performed in analog version on a Siemens Mammomat Nova 3000 X-ray unit with the following exposure parameters: tube voltage of 35 kV, tube current of 10 mA, exposure time of 100 ms, 0.3 mm lamp focus, and Mo/Mo filtration. Each of the implants was shown in two projections: antero-posterior and lateral.

On the obtained images the visibility and the number and location of the implants in the femoral bones of the rabbits were assessed. For implantation evaluation, the following parameters were identified and analyzed: the presence of osteolysis and osteosclerosis around the implants; closure of the borehole in the femoral bone through so-called ‘bone cap’ creation; bone canal callus filling; the quality of bone trabeculae; and the presence of periosteal reactions.

### 3.6. Histological Studies

The femoral fragments with the implants were fixed for 72 h in 10% aqueous formic formaldehyde in phosphate buffer. Then, the samples were decalcified in a solution of formic acid and hydrochloric acid, dehydrated in acetone (at a temperature of 56 °C), X-rayed in xylene at room temperature, and embedded in paraffin blocks. With the use of a microtome (Lecica Microsystems Inc., Bannockburn, IL, USA) thick sections of approximately 4 μm were cut. The prepared samples were dyed with haematoxylin and eosin (HE) by the Van Gieson method (VG) to differentiate stromal connective tissues. Histological specimens were evaluated under a light microscope (Olympus BX43, Olympus, Tokyo, Japan) using a computer program for analysis and image acquisition (cellSens Standard, Olympus).

The qualitative assessment included: (1) the type of tissue filling the area of the implant in the intraosseous defect; (2) the amount of resorption of the biomaterial; (3) the method of integrating the alloplastic material with the surrounding tissues filling the intraosseous defect; and (4) the presence of cells indicating an inflammatory process. The quantitative assessment included, first, the relative area of newly formed bone and, second, the surface of the relatively non-resorbed implanted material. The relative surfaces examined were presented as a percentage of the total surface area of the test for each group.

The degree of degradation of the fibrous implants examined was assessed on the basis of the measurements of the diameter of individual circular filaments. The *t*-test was used for the statistical evaluation.

## 4. Results

### 4.1. The Evaluation of Allergenic Effect

**Figure 2:**
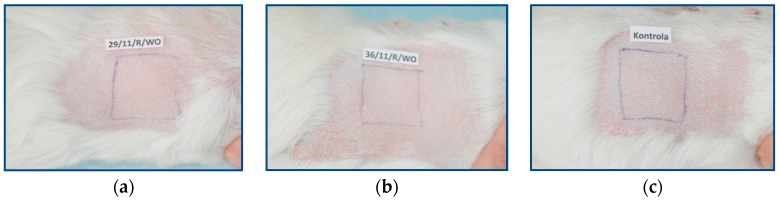
Animal skin at the application site of extracts from samples of the following materials: (**a**) PLGA + IGF1, (**b**) PLGA/PHB + IGF1, and (**c**) control.

The evaluation of the skin lesions was performed 24 h and 48 h after the removal of test samples. The examination sites were classified according to the Magnusson and Kligman scale. Throughout the duration of the experiment, the animals were under constant veterinary care. The monitoring included a general inspection of the animals, with a particular emphasis on body weight. At 24 h and 48 h after the removal of the test samples from any of the tested guinea pigs, there was no allergic reaction. During the challenge phase, normal appearance was found on the animals’ skin where the extracts from PLGA + IGF1 and PLGA/PHB + IGF1 were applied, and the skin did not differ from the skin treated with the control extracts (Figure 2).

In both the tested and control groups, the animals’ health did not deviate from the norm. The animals showed normal weight gain, which, at the end of the experiment, was 30 g for the PLGA + IGF1 group, 27 g for the PLGA/PHB + IGF1 group, and 37 g for the control group.

### 4.2. Evaluation of Local Reactions after Implantation

Up to 48 h after the surgery and implantation, some animals showed a decreased appetite, but, on the third day, food consumption returned to normal and was approximately 50 to 70 g per day. In each group of rabbits, the animals’ condition did not deviate from the norm throughout the course of the research. The animals retained active and passive mobility of the hip joints, and the surgical wounds healed properly. In individual animals in both the experimental and control groups, the area around the hip joint was slightly enlarged at one and two months after the operation but later returned to normal. All animals survived until the scheduled postmortem examination.

### 4.3. Macroscopic Evaluation

In all animals examined postmortem, the postoperative skin wounds had healed properly. The soft tissues surrounding the body of the femur had a normal appearance. The macroscopic images of all the examined and control groups were similar, and the found differences are underlined below. In individual subjects in both the experimental and control groups in the early period (one and two months after the implantation), an above-average serous effusion at the wound area was observed at the site of implantation. The macroscopic examination revealed a normal shape of the femur. Bone defects fitted with implants in smaller and larger trochanters that were covered with periosteum remained visible in the early periods and, in later periods, were visualized after dissection of the soft tissue and periosteum covering the site of implantation. Concerning the size of trochanters with implants in individual animals, the materials containing IGF1 were slightly increased compared to the control group. At all the examination dates, the femur implants were invisible in longitudinal cross-sections of the tissue of macroscopically normal appearance.

**Figure 3:**
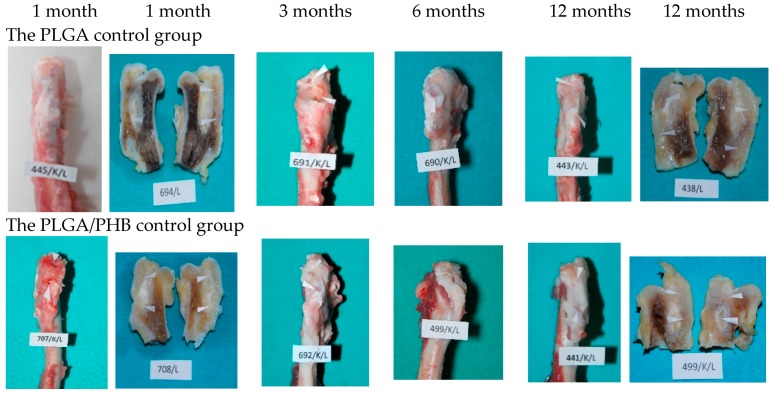

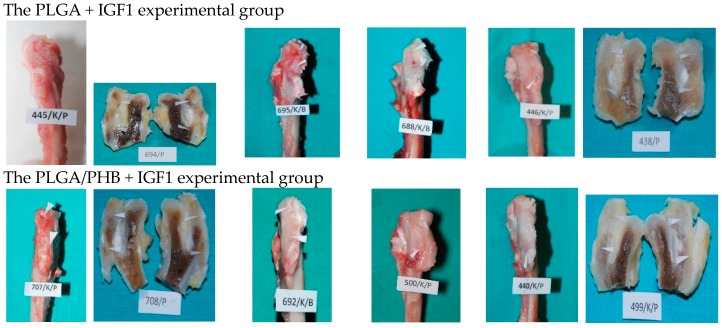
Macroscopic images of femoral trochanters and longitudinal sections at one, three, six, and 12 months after implantation. Locations of the implants are marked with arrows.

Photographs of the samples of the bones at the implantation sites are shown in Figure 3.

### 4.4. Radiological Evaluation

**Figure 4:**
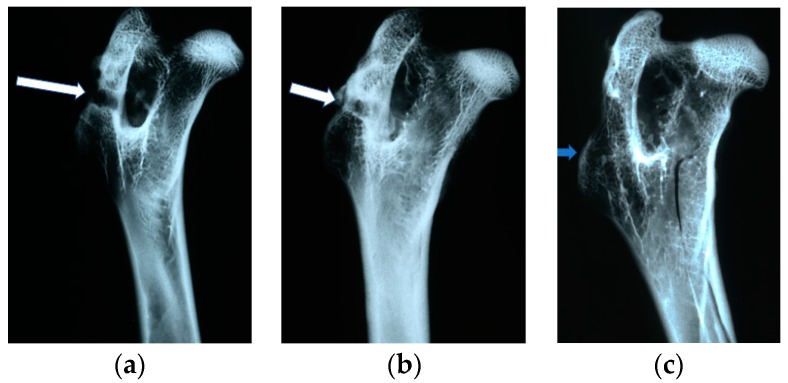
Radiological images after implantation of the tested materials: (**a**) 1 PLGA after one month. Visible implantation borehole. No callus. Slight periosteum around the implant. (**b**) PLGA after two months. Implantation borehole closed with a bone cap. Channel partially filled with callus. (**c**) PLGA after 12 months. Implantation channel invisible, overgrown with bone trabeculae.

**Figure 5:**
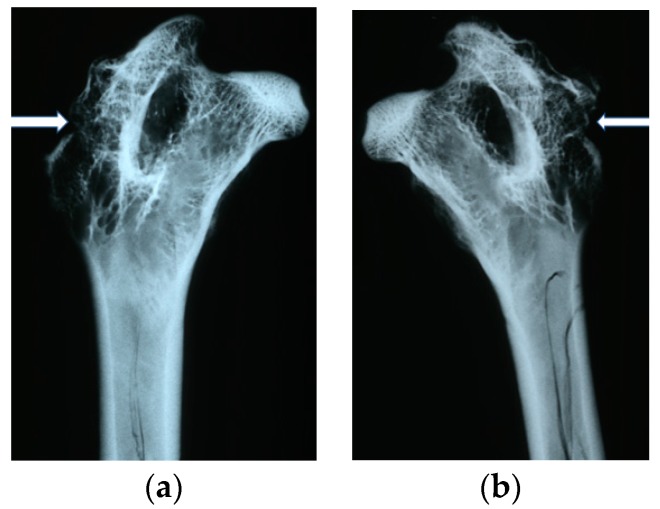
Radiological images after implantation of the tested materials: (**a**) PLGA after six months. Traces of the implant channel are visible, filled with thin bone trabeculae. The implant channel is closed with a thin bone cap. (**b**) PLGA + IGF1 after six months. In contrast to the opposite side, the implant channel is invisible, filled with numerous thick bone trabeculae, and there is a thicker bone cap at the point of the borehole.

In X-ray imaging, all the implants remained translucent and invisible. At the first month after implantation, it was observed that the implant canals with implanted materials, PLGA + IGF1 and PLGA/PHB + IGF1, were partially filled with callus, which was partially sclerotic. In the implantation locations for the control materials, PLGA and PLGA/PHB, only traces of calluses were observed. All openings at the implantation sites were open. After two months, a continuation of filling with calluses was observed in the implant canals of the PLGA + IGF1 and PLGA/PHB + IGF1 samples. The implant holes remained open in the PLGA + IGF1 samples, while, in the PLGA/PHB + IGF1 samples, most holes were closed with osseous lamina. In individual control samples (PLGA/PHB), a marginal amount of osteosclerosis or osteolysis was observed. After three months, the implant canals were closed entirely or, in individual samples, partially closed with spongy calluses. In the PLGA implant materials, the implant canals were closed, except in some individual samples, in which the canals were partially filled with calluses from the bottom, while the entrances remained open. Six months after implantation, the implant canals were filled with spongy calluses. In individual control samples, trace osteolysis was recorded. All implant holes were closed by osseous lamina. In subsequent post mortem examinations (after nine to 12 months), the macroscopic images were similar. There were no periosteal reactions in the autopsy period. Radiographic images of changes within the groups are presented in Figure 4. The persistent differences between the control groups and the IGF1 tested groups are shown in Figure 5.

### 4.5. Microscopic Evaluation

#### 4.5.1. Histological Evaluation after Implantation PLGA + IGF1 (Experimental Group) and PLGA (Control Group)

**Figure 6:**
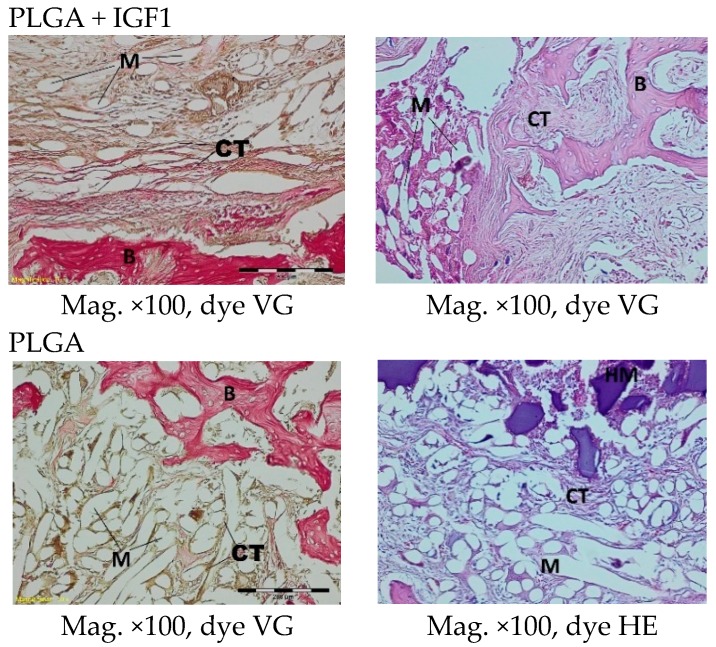
Microscopic images one month after the implantation of the experimental PLGA + IGF1 and control PLGA. M-material, CT-connective tissue, B-bone tissue, BM-bone marrow, HM-homogeneous masses. PLGA + IGF1—left image: the edge of the implant with the wide bands of connective tissue between the fibers of the material is visible; right image: at the edge of the implant with a forming of trabeculae is visible. PLGA—left image: the edge of the implant with the thin bands of connective tissue is visible; right image: the homogenous masses in the centre of implant.

In histological specimens in both the experimental and control groups at the first month after implantation, some multifibre grafts or oval spaces after the washed material were visible in the spongy bone and were surrounded by loose connective tissue, characteristic of inflammatory granulation, which penetrates between the individual fibres. In the described bands, fibroblasts, fibrocytes, and a significant number of thin-walled blood vessels could be distinguished. The bands of connective tissue in the experimental group were wider than in the control. In individual cases, in the centre of the specimens, some homogeneous masses with no apparent accumulation of inflammatory cells were visible. Within the specimens from bone with PLGA + IGF1 implants, some trabeculae (less numerous than in the group with PLGA/PHB + IGF1) were visible at the edges, and, in the bone tissue surrounding the implant, some reconstruction and osteoblast activity were visible (Figure 6).

**Figure 7:**
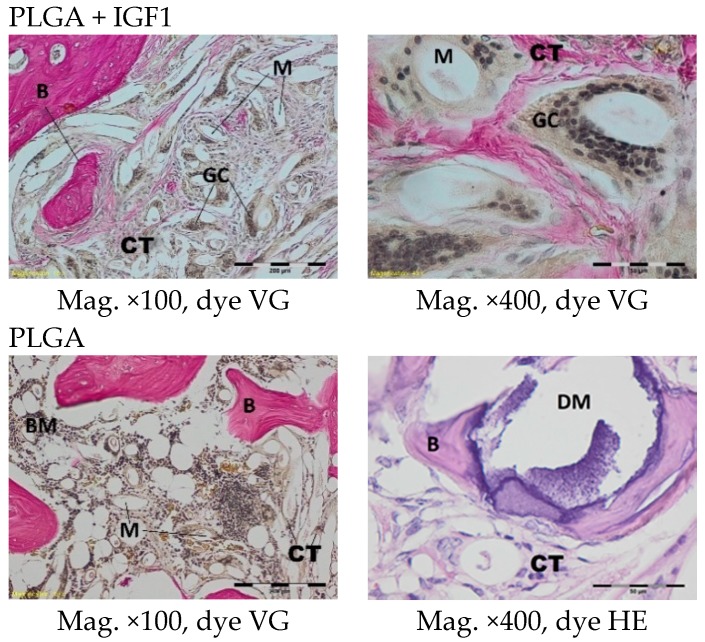
Microscopic images three months after the implantation of the experimental PLGA + IGF1 and control PLGA. M-material, CT-connective tissue, B-bone tissue, BM-bone marrow, GC-giant cell, DM-decomposition of material. PLGA + IGF1—left image: the edge of the implant with some trabeculae and fibrous connective tissue; right image: around the individual implant filaments the giant cells are visible. PLGA—left image: the edge of the implant with residues of material in the bone marrow; right image: breakdown of the fragments of the implants.

The histological images of the spongy bone tissue three months after implantation revealed some implant fibres in both the experimental and control materials, separated by a broad region of connective tissue that had a fibrous structure on the outside and was loose in the immediate vicinity of the implant. In the region around the implant, some trabeculae were visible. In addition, in both the experimental and control groups, giant cells were observed around the filaments of the implant. Some breakdown of the fragments of the implants in the produced trabeculae was observed (Figure 7).

**Figure 8:**
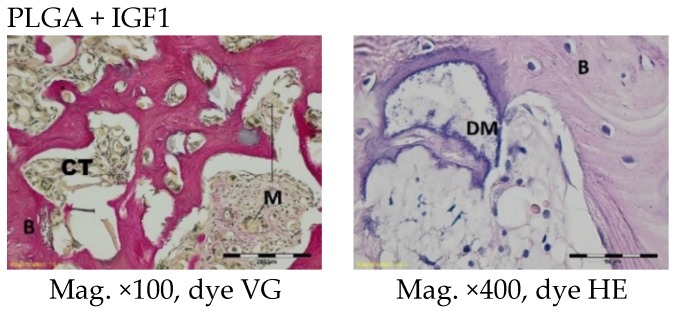

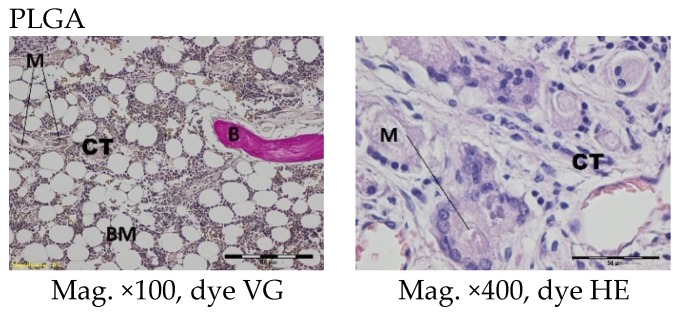
Microscopic images six months after the implantation of the experimental PLGA + IGF1 and PLGA control. M-material, CT-connective tissue, B-bone tissue, BM-bone marrow, GC-giant cell, DM-decomposition of material. PLGA + IGF1—left image: spongy bone in the edge of the implant; right image: focal disintegration of the material is visible. PLGA—left image: the residues of materials in the bone marrow are visible; right image: granulation tissue around the material.

Spongy bone was found at the implantation sites in the histological specimens after six months. Residues of materials in the form of small fibrils were present in the bone marrow, surrounded by granulation tissue with inflammatory characteristics. In the experimental group, some focal disintegration of the material embedded in the trabeculae was observed (Figure 8).

**Figure 9:**
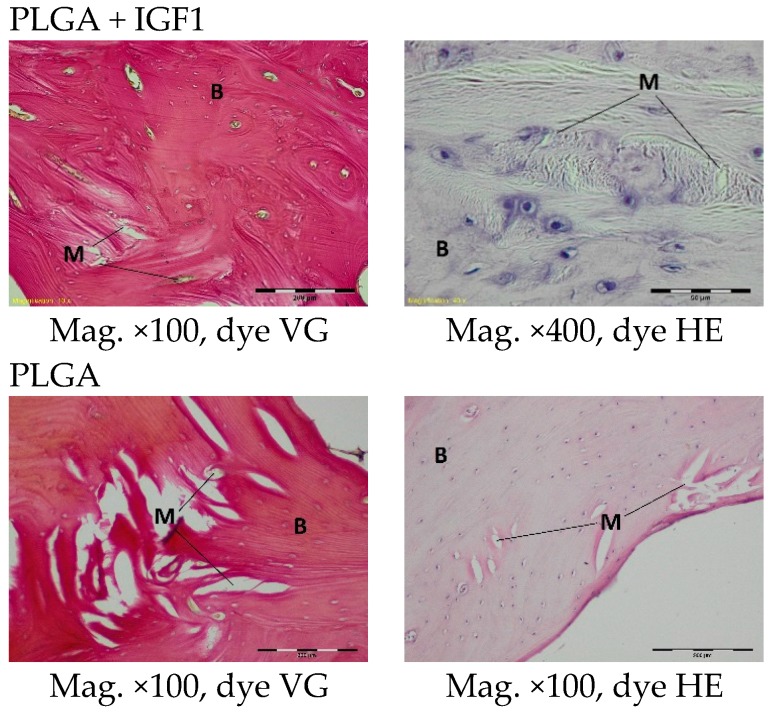
Microscopic images nine months after the implantation of the experimental PLGA + IGF1 and control PLGA. M-material, B-bone tissue. PLGA + IGF1—left image: on the left-spongy bone in the edge of the implant is visible; right image: the remains of the implant material and the traces of bone remodelling. PLGA—left image: the edge of the implant; right image: the material in the lamellar bone tissue.

**Figure 10:**
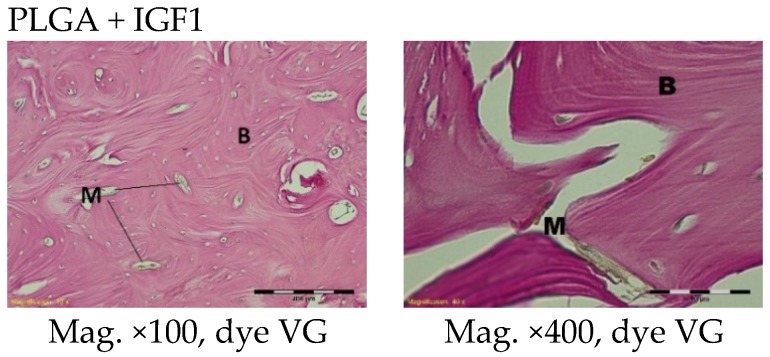

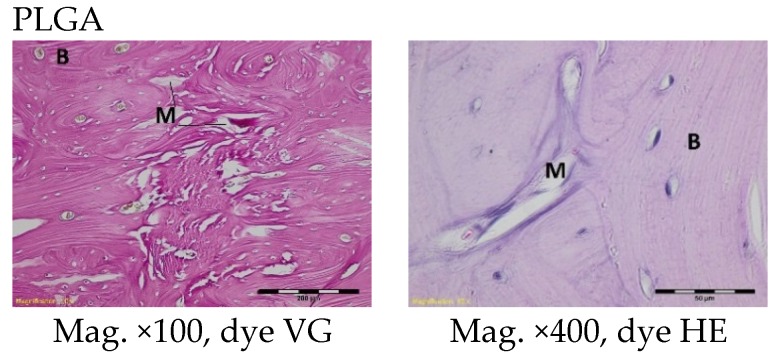
Microscopic images 12 months after the implantation of the experimental PLGA + IGF1 and control PLGA. M-material, B-bone tissue. PLGA + IGF1—left image: the edge of the implants with the spongy bone; right image: the residue of fibre in trabeculae. PLGA—left image: the edge of the implant; right image: in the trabeculae the residue of material is visible.

At nine and 12 months after implantation, the macroscopic images were similar and are therefore described together. At the site of the implantation of the experimental and control implants, spongy bone tissue was present. In isolated places in the trabeculae, some traces of bone remodelling and osseous bone (textus osseous rudifibrosus), as well as small remains of the implant material, were found (Figure 9 and Figure 10).

#### 4.5.2. Histological Evaluation after the Implantation of PLGA/PHB + IGF1 (Experimental Group) and PLGA/PHB (Control Group)

**Figure 11:**
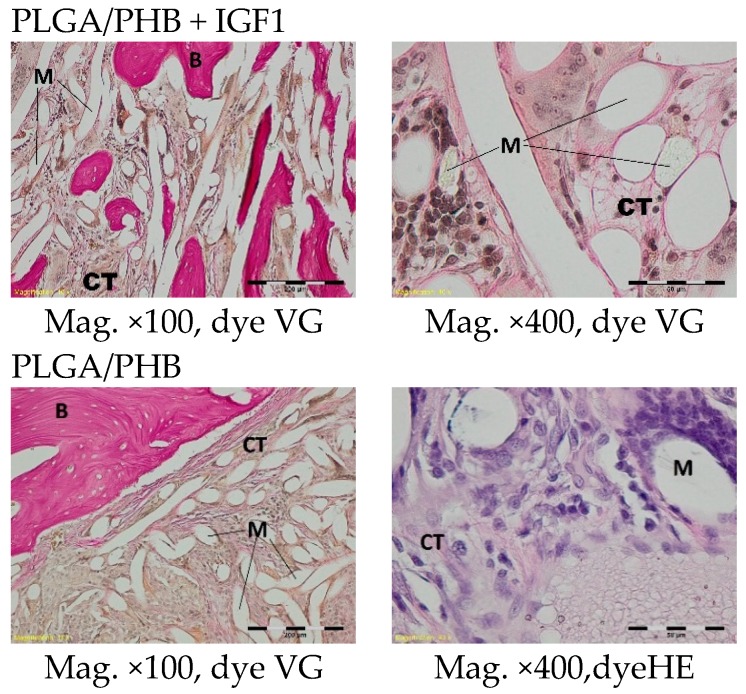
Microscopic images of bone tissue one month after the implantation of the experimental PLGA/PHB + IGF1 and control PLGA/PHB. M-material, CT-connective tissue, B-bone tissue. PLGA/PHB + IGF1—left image: the edge of the implant with single trabelcure is visible; right image: the connective tissue in the centre of implant is visible. PLGA/PHB—left image: the edge of the implant with connective tissue is visible; right image: the connective tissue in the centre is visible.

Histological images of the tissue one month after implantation showed multifilament experimental and control implants, as well as the connective tissue that surrounded the thin strands of individual filaments. In the area surrounding the implant, it was possible to observe fibroblasts and fibrocytes forming multinucleated macrophages and thin-walled blood vessels. At the points of contact with the bone marrow, mainly connective tissue was visible. In individual cases in both groups, in the central part of the implant, homogeneous masses were observed, with no visible accumulation of inflammatory cells. In the experimental group, within the peripheral part of the implant, a single trabeculae was present, and the surrounding tissue was reconstructed, with osteoblast activity visible (Figure 11).

**Figure 12:**
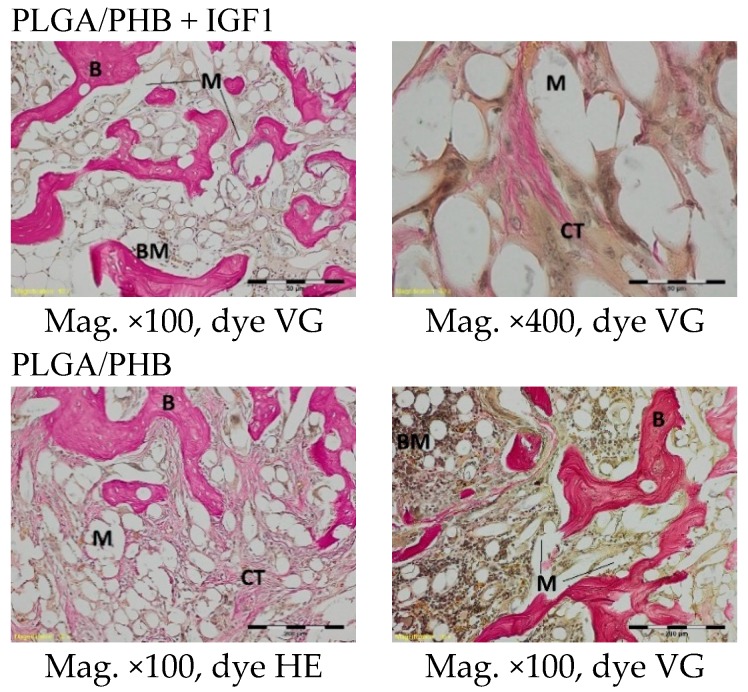
Microscopic images of bone tissue two months after the implantation of the experimental PLGA/PHB + IGF1 and control PLGA/PHB. M-material, CT-connective tissue, B-bone tissue, BM-bone marrow. PLGA/PHB + IGF1—left image: the edge of the implant, a trabeculae of spongy bone tissue is visible; right image: the loose and fibre connective tissue in the centre is visible. PLGA/PHB—left image: the edge of the implant with some trablecure, right image: the connective and bone tissue in the edge of the implant are visible.

A microscopic examination of the bone two months after implantation showed loose and fibrous connective tissue surrounding the implant filaments in the centre of both the experimental and control implants. At the outer part of the implant, a trabeculae of spongy bone tissue was visible. In the immediate vicinity of the implant fibrils, some multinucleated macrophages (giant cells) associated with the degradation of the implant were observed. In the tissue surrounding the implant, it was possible to observe fibroblasts, fibrocytes, and mononuclear inflammatory cells forming multinucleated macrophages and collagen fibres. In individual cases, in the centres of the specimens, homogeneous masses with no apparent accumulation of inflammatory cells were visible (Figure 12).

**Figure 13:**
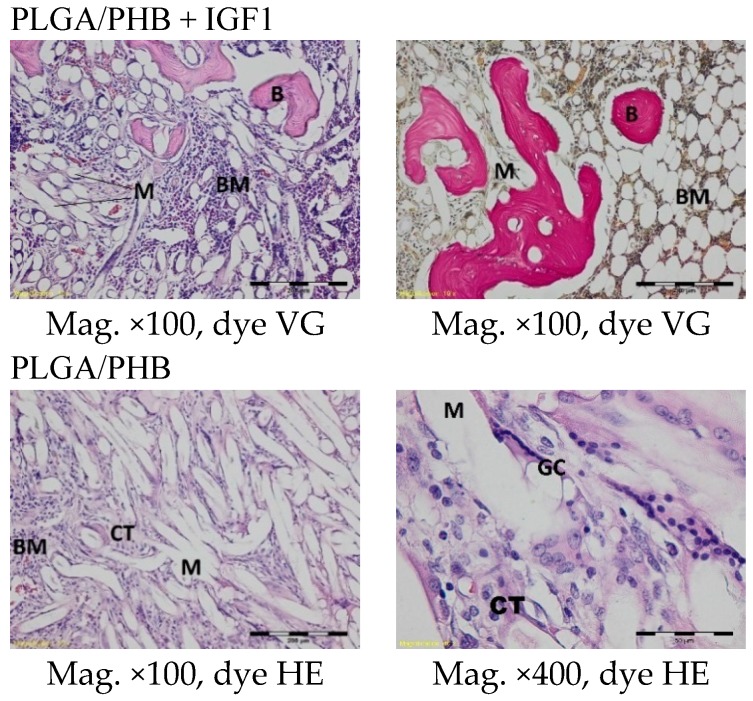
Microscopic images of bone tissue three months after the implantation of the experimental PLGA/PHB + IGF1 and control PLGA/PHB. M-material, CT-connective tissue, B-bone tissue, BM-bone marrow, GC-giant cell. PLGA/PHB + IGF1—left image: the material and connective tissue in the centre of the implant is visible; right image: trabeculae in the edge of the implant. PLGA/PHB—left image: the centre of the implant with visible fibrous structure; right image: the filament of material surrounded by the connective tissue and giant cells

In the centre of the experimental and control implants, loose connective tissue surrounding the individual filaments was observed three months after implantation. In the outer part of the implant in the experimental group, bone trabecular tissue was visible. In the immediate vicinity of the implant fibrils, the formation of a few giant cells associated with the degradation of the implant was observed. In the tissue surrounding the implants, it was possible to distinguish fibroblasts, fibrocytes, mesenchymal cells, a few mononuclear inflammatory cells, and thin-walled blood vessels (Figure 13).

**Figure 14:**
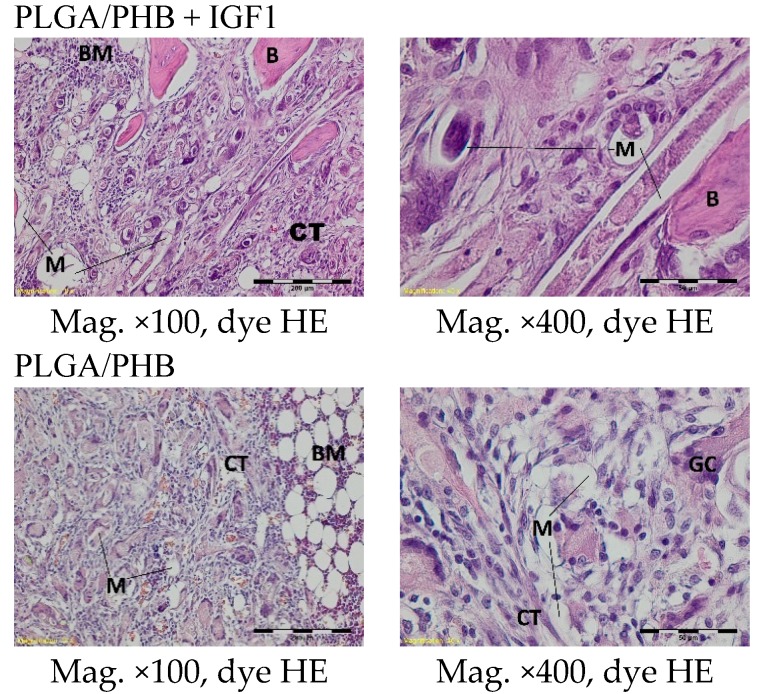
Microscopic images of bone tissue six months after the implantation of the experimental PLGA/PHB + IGF1 and control PLGA/PHB. M-material, CT-connective tissue, B-bone tissue, BM-bone marrow, GC-giant cell. PLGA/PHB + IGF1—left image: the residues of material in bone marrow are visible; right image: the remains of the fibres surrounded by connective tissue are visible. PLGA/PHB—left image: the residues of material in the bone marrow; right image: loose and fibrous connective tissue around the material in the centre of implant is visible.

Compared to previous research, a degradation of fibrils was observed in the histological specimens six months after implantation in both the experimental and control implants. Around and inside the implant fibrils, mesenchymal cells and multinucleated macrophages were visible. In the centre of the implants, there was loose and fibrous connective tissue, with numerous thin-walled blood vessels, characteristic of the inflammatory granulation tissue that surrounded the individual filaments. In the outer part of the implant, some trabeculae of the spongy bone tissue were visible. In the immediate vicinity of the implant, fibrils and giant cells associated with the degradation of the implant were present. In the area surrounding the implant, there were fibril tissues, fibroblasts, fibrocytes, and thin-walled blood vessels (Figure 14).

**Figure 15:**
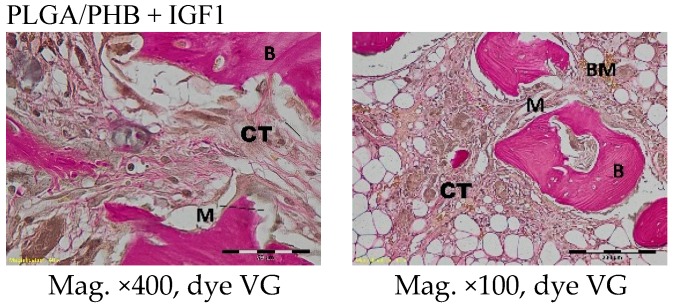

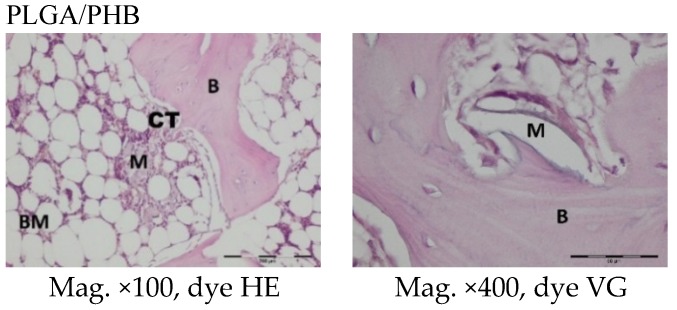
Microscopic images of bone tissue nine months after the implantation of the experimental PLGA/PHB + IGF1 and control PLGA/PHB. M-material, CT-connective tissue, B-bone tissue, BM-bone marrow .PLGA/PHB + IGF1—left image: in the immediate vicinity of the irregular residues of material the loose connective tissue is visible: right image: the residues of material in the spongy bone. PLGA/PHB-on the left- the residues of material in the bone marrow of spongy bone; right image: the remains of the material in the lamellar bone in the edge of the implant.

In the histological specimens of the tissue nine months after implantation, there were some trabeculae of lamellar bone tissue and low quantities of loose and fibrous tissue in the bone marrow in the immediate vicinity of the irregular implant material residues within the implant area. The amount of implant material was smaller than in previous studies. Around and within the fibrils of the implant (material residues), mesenchymal cells and multinucleated macrophages were observed (Figure 15).

**Figure 16:**
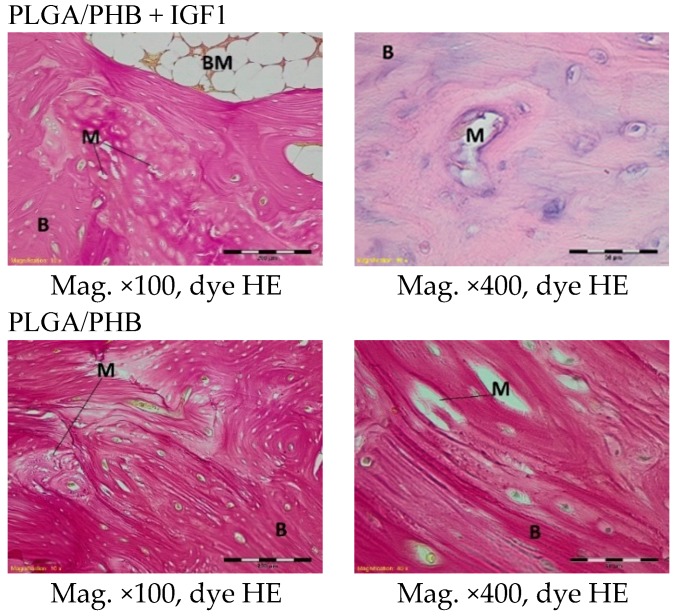
Microscopic images of bone tissue 12 months after the implantation of the experimental PLGA/PHB + IGF1 and control PLGA/PHB. M-material, CT-connective tissue, B-bone tissue, BM-bone marrow. PLGA/PHB + IGF1—left image: the edge of the implant with spongy bone is visible; right image: the implant residues in the bone tissue are visible. PLGA/PHB—left image: the edge of the implant with bone tissue; right image: the small remnants of the implant are visible.

A microscopic examination of the tissues collected twelve months after implantation revealed spongy bone tissue at the implantation site in both the experimental and control groups. Within the trabecular bone made of lamellar bone, new reconstructions with small remnants of the implant were visible (Figure 16).

### 4.6. Histological Quantitative Assessment

**Figure 17:**
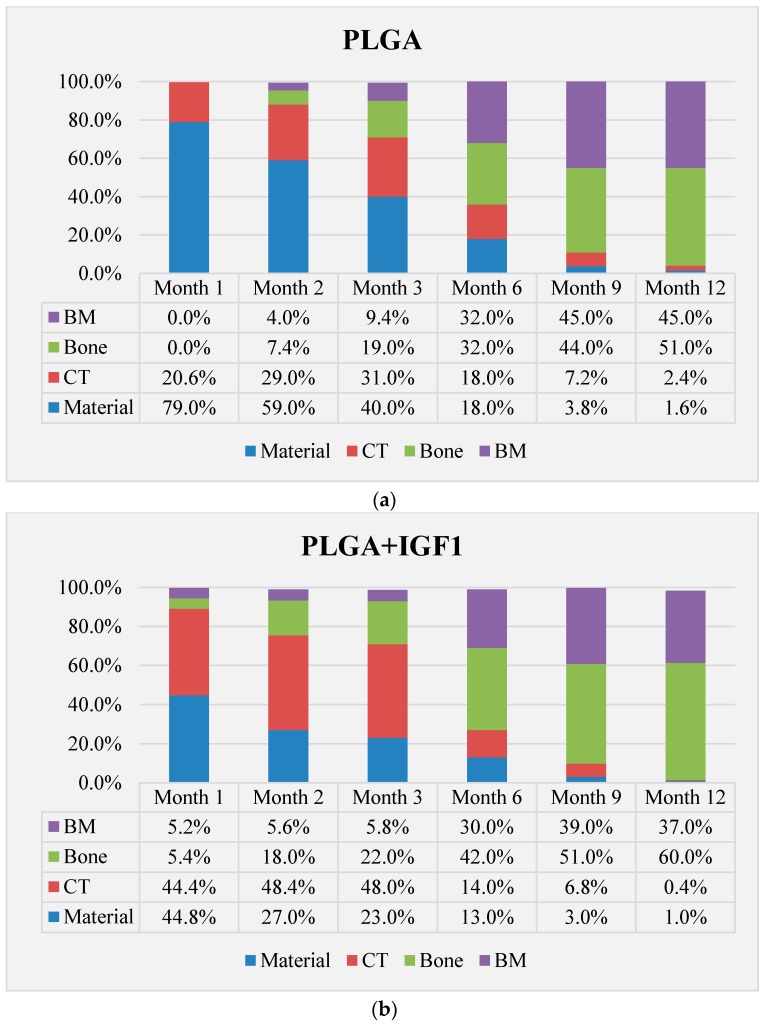

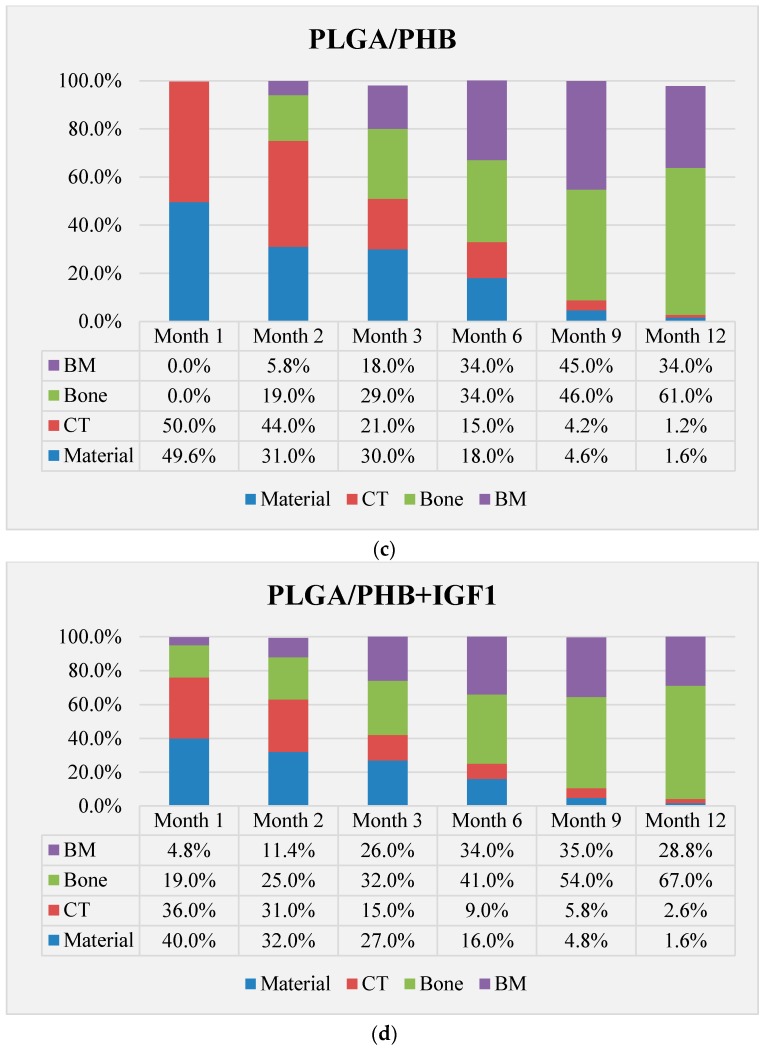
Relative average values of bone tissue and biomaterial surfaces in the treated bone defects after one, two, three, six, nine and 12 months. Amounts calculated for places of implantation of: (**a**) PLGA, (**b**) PLGA + IGF1, (**c**) PLGA/PHB, and (**d**) PLGA/PHB + IGF1.

In the histological evaluation of the implants in the early period of bone healing, different dynamics and different amounts of connective and bone tissue were observed. After one month, the highest amounts of bone tissue were visible in the area of PLGA/PHB + IGF1, followed by the PLGA + IGF1 and the control group. This trend lasted throughout the 12-month observation period. The PLGA/PHB implants stimulated greater proliferation of connective tissue in the first few months and increased bone tissue in the later period compared to PLGA implants (Figure 17a–d).

### 4.7. Degradation of the Materials after Implantation

**Figure 18:**
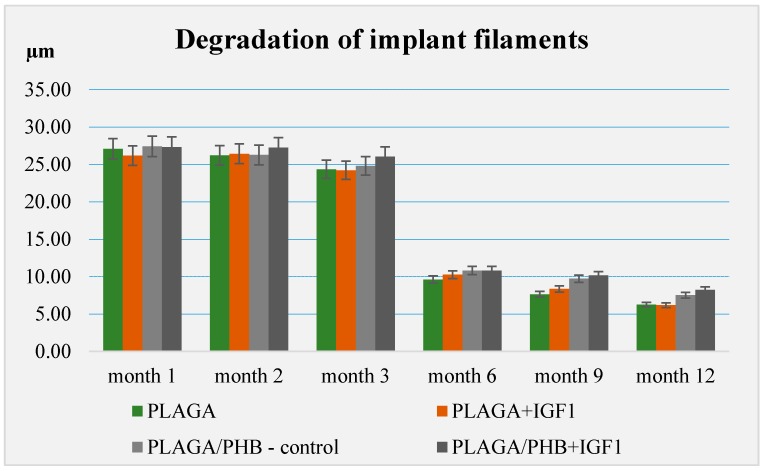
The degree of fibre degradation in the experimental implants (PLGA + IGF1, PLGA/PHB + IGF1) and in the controls (PLGA, PLGA/PHB).

In all the experimental groups and the control group, a significant decrease in the diameter of individual fibres was observed between three and six months (* *p* < 0.05). In the long term, slightly lower values were observed for PLGA and PLGA + IGF1 than for PLGA/PHB and PLGA/PHB + IGF1 (Figure 18).

## 5. Discussion

Biodegradable fibrous products based on a newly developed polymer with reduced toxicity, PLGA with poly(hydroxybutyrate) (PHB), were designed and prepared. The newly developed implants had a porous structure and contained microspheres with growth factor IGF1, as well as Hap nanoparticles, to support regenerative processes and increase bioactivity. The use of multilayer systems allowed the creation of a material with varied porosity. Combining macroporous and mesoporous layers increased the total area of the pores. The increase in average pore size resulted from the process of connecting the two layers during needle-punching. This significant increase in apparent density suggests increased packing of the fibres in the process of combining layers, as well as an association with the introduction of microspheres with growth factors, which partially fill the pores of the material.

In the evaluation of the biocompatibility and bioactivity of the porous, fibrous bone implants of experimental PLGA + IGF1 and PLGA/PHB + IGF1 and control PLGA and PLGA + PHB, tests for allergenic reaction and tests for local bone tissue response after implantation for a period of one, two, three, six, nine, and 12 months were carried out. In the scheduled postmortem periods, macroscopic and radiological evaluations were performed, followed by microscopic histological evaluations of the healing process and the degradation time of the implanted materials. Our research revealed similar and correct clinical pictures for all types of implants. Every animal in both the experimental and control groups survived. The surgical wounds were healed by first intention. The animals retained active and passive mobility of the hip. Individual animals in the early period, one to two months after surgery, demonstrated slightly larger surroundings of the hip joint. During later periods, no change was noticed. Macroscopically, the soft tissues around the hip, following the implantation of all investigated implants, were correct and similar. During autopsy, moderate amounts of colourless exudate around the implant were observed in both the control group and the experimental group for individually tested animals one month after implantation. The macroscopic images of the experimental group and the control group in the early period showed the locations of the implantation on the trochanters’ surface. In subsequent periods, the implants were covered by periosteum and were barely visible. For individual animals from the experimental group (with a supplement of IGF1), a slight increase of femur trochanters was found in the macroscopic images; for the other animals, the shape and size of the trochanters were comparable to those in the control group. In X-ray imaging, all the implants remained translucent and invisible. Upon examination one month after implantation, the canals with PLGA + IGF1 and PLGA/PHB + IGF1 were partially filled with calluses, while, in those with control materials, only traces of callus were visible. After two months the implant holes remained open in the PLGA + IGF1 samples, while, in the PLGA/PHB + IGF1 samples, most of the implant holes were closed with osseous lamina. At later observations in all the groups the implant canals were filled with spongy calluses and closed by osseous lamina, partially after three months and entirely after six, nine, and 12 months. Compared to the control samples, those implanted with the tested materials were filled with thicker bone trabeculae.

In the histological studies, in the spongy bone tissue at the implantation site for all implants (both experimental and control), within one month after implantation, a narrow band of loose connective tissue, characteristic of inflammatory granulation tissue, surrounded the implant, and the individual filaments of the implant were revealed. In the centre of the implantation site in individual cases, within one to two months after implantation, small amounts of homogeneous masses (corresponding to exudate) were visible. In addition, in the experimental groups (with IGF1), newly developed trabeculae could be observed around the implants within one month after implantation. In the surrounding tissue, increased osteoblast activity was observed. The formation of trabecular bone tissue after only one month has also been described in studies following the augmentation of animal mandible defects with xenogenic implants based on bovine bone with collagen-containing polypeptide growth factors [7,33,34]. 

More intensive expansion of the trabeculae was observed within one to two months after implantation for experimental group PLGA/PHB + IGF1 than for experimental group PLGA + IGF1. Furthermore, the implanted materials gradually degraded while the loose and fibrous connective tissue was undergoing replacement by the spongy bone tissue. In the immediate vicinity of the residues of the implants, the presence of cell-rich connective tissue, including very few inflammatory and mesenchymal cells, was observed. Excessive PLGA process biodegradation of the material from both the experimental and control groups was observed at three months after implantation, with observed activity of multinucleated macrophages (giant cells). Some filament residues of PLGA were visible up to six months, while, after 12 months, they were hardly noticeable. Experimental and control implants of PLGA + PHB later showed insignificant increases in biodegradation, and filament residues were also visible up to nine to 12 months after implantation. In all the groups, there were statistically significant reductions in the diameters of individual filaments between three and six months.

Focally, in the newly produced bone, the decomposition of implant material incorporated into bone lamellae was observed for the control group, PLGA, after three months and in the experimental group, PLGA + IGF1, after six months. The PLGA/PHB + IGF1 implants showed a higher osteostimulation property in the early period and degraded later compared to PLGA + IGF1.

Similar observations of the beneficial effects of using a PHB membrane and the increased filling of defects with bone tissue compared to the control group were also found in other studies [35,36]. The transient presence of the giant cells’ reaction related to the biodegradation of the polymer was observed in histopathological studies by other researchers; e.g., in the experiment in which patches of PHB were applied, and in which, after 12 months, fragments of material and associated giant cells were found [37]. On the basis of quantitative histological tests performed at all the observational time points, the experimental group with IGF1 demonstrated increases in the relative surface area of bone trabecular filling the intraosseous defects compared with the control group.

The results obtained are consistent with the data, which suggests that IGF1 released from the bone matrix during bone resorption produces an osteogenic micro-environment and induces the differentiation of recruited mesenchymal stem cells (MSCs) to obtain new bone. Next, the authors postulate that the primary function of IGF1 in bone matrix is to maintain bone mass and skeletal homeostasis during bone remodelling [21]. The benefits of using IGF1 are also reported by other authors [38]. A similar effect was reported in an animal study focused on the histological characteristics of the early osseointegration of implants with or without the addition of platelet-rich plasma (PRP) or combined platelet-derived growth factor (PDGF)/IGF1. The results showed greater new bone deposition in animals in which PDGF/IGF1 was added to the implants, compared to animals treated with PRP or to the controls [19]. Other studies have indicated that applying polypeptide growth factors in the early period of bone healing has a favourable effect [7].

Moreover, in our research, in the period up to three months after implementation, histological observation of the implantation sites of the experimental and control materials revealed the very good condition of the bone marrow.

In a study of the sensitisation effect, the animals’ skin, at the application site of the experimental extracts of PLGA + IGF1 and PLGA/PHB + IGF1, had a regular appearance compared to the control group. In both the experimental and control groups, the animals’ health did not deviate from the norm. The animals showed normal increases in body weight, and the gender of the animals had no effect on the results.

## 6. Conclusions

Studied bone implants produced from the newly developed zirconium-based copolymer of lactide and glycolide, with and without insulin-like growth factor (PLGA + IGF1 or PLGA, respectively), as well as the implant produced from the blend of this copolymer with poly([R,S]-3-hydroxybutyrate), reinforced or not with IGF1 (PLGA/PHB + IGF1 or PLGA/PHB, respectively), did not cause negative changes in the health status of animals in clinical trials up to 12 months after implantation in the experimental or control groups.

Macroscopic evaluation of the hip joint surroundings in the early stage, i.e., one to two months after implantation, showed a small amount of colourless and serous fluid at the implantation site in individual animals. At later time points, there was no exudation, and the observed image was normal. In the macroscopic evaluation, the femurs with implants were normal, and only in individual animals from the experimental group (with the addition of IGF1) were the trochanters slightly enlarged.

All materials only weakly induced an inflammation reaction, leading to the formation of increased quantities of cancellous and lamellar bone tissue and good bone marrow condition at the site of implantation within nine to 12 month. The tested materials with IGF1 induced a greater percentage of bone mass than the control implants. In the early period of bone healing, the experimental implant PLGA/PHB + IGF1 revealed higher osteostimulation properties and degraded later in comparison to the PLGA + IGF1 samples. All the materials gradually degraded. The most rapid statistically significant degradation occurred between three and six months. It should be noted that the designed porous structure of the material is beneficial for healing tissue and allows for free material overgrowth of cells. The structure of the implant, bioactive substances, and especially IGF1 allow for the acceleration of tissue regeneration. The obtained results of the maximisation test for a sensitisation effect allow us to conclude that samples of the materials, PLGA + IGF1 and PLGA/PHB + IGF1, did not cause allergic reactions on the skin of guinea pigs.
